# Expression of gamma-aminobutyric acid type A receptor subunit π and minichromosome maintenance-3 in breast cancer patients treated with neoadjuvant chemotherapy: a target molecule or a proliferation marker?

**DOI:** 10.1590/1806-9282.20250507

**Published:** 2025-10-17

**Authors:** Yakup İriagac, Meltem Oznur, Eyyup Çavdar, Kubilay Karaboyun, Seray Saray, Okan Avcı, Erdoğan Selçuk Şeber

**Affiliations:** 1 University of Health Sciences, Balikesir Ataturk City Hospital, Department of Medical Oncology – Balıkesir, Turkey.; 2 Tekirdag Namik Kemal University, Department of Pathology, Faculty of Medicine – Tekirdag, Turkey.; 3 Adiyaman University, Research and Training Hospital, Faculty of Medicine, Department of Medical Oncology – Adıyaman, Turkey.; 4 Agri Ibrahim Cecen University, Training and Research Hospital, Faculty of Medicine, Department of Medical Oncology – Ağrı, Turkey.; 5 Tekirdag Namik Kemal University, Faculty of Medicine, Department of Medical Oncology – Tekirdağ, Turkey.

**Keywords:** GABRP, MCM3, Breast cancer, Neoadjuvant, Targeted therapy

## Abstract

**OBJECTIVE:**

The receptors and cell membrane channels found in breast cancer in high numbers have been promising for drugs in the past and in the future. In addition, analyses of proliferation indices play an essential role in predicting the success of these treatments.

**METHODS:**

Gamma-aminobutyric acid type A receptor subunit π and minichromosome maintenance-3 expressions were studied by immunohistochemical staining from tissue samples obtained by trucut biopsy from epidermal growth factor receptor 2-negative breast cancer patients who received neoadjuvant chemotherapy.

**RESULTS:**

The study included 101 patients. Almost 96.0% of the breast tumor tissues expressed gamma-aminobutyric acid type A receptor subunit π. The median value for gamma-aminobutyric acid type A receptor subunit π was calculated as 30% (19 patients) and the median for minichromosome maintenance-3 as 80% (13 patients). When neoadjuvant chemotherapy response was evaluated, pathological complete response was achieved in 23.8% of patients (n=24). Pathological complete response was achieved in 16.4% of patients for hormone receptor positivity and in 38.2% of patients for triple-negative breast cancer. When the factors predicting pathological complete response were evaluated with univariate analysis, age (p=0.044), molecular subtype (p=0.017), menopausal status (p=0.032), Ki-67 increase (p<0.001), estrogen receptor decrease (p<0.006), progesterone receptor decrease (p<0.002), and grade (p<0.001) predicted treatment response. There was no relationship between gamma-aminobutyric acid type A receptor subunit π (p=0.945) and minichromosome maintenance-3 (p=0.985) and treatment response.

**CONCLUSION:**

Our study found that gamma-aminobutyric acid type A receptor subunit π is highly expressed in breast cancer cells but absent in normal breast tissue, making it a potential therapeutic target. Additionally, we determined that minichromosome maintenance-3 is not a reliable proliferation index or predictor of treatment response.

## INTRODUCTION

According to current data, breast cancer is the most common type of cancer and the second most common cause of cancer-related deaths in women^1^. One of the preferred treatment options for locally advanced breast cancer is neoadjuvant chemotherapy (NAC). This approach increases the feasibility of breast- and axilla-conserving surgery while also providing the opportunity to evaluate the sensitivity of the tumor to chemotherapy^2^. Large-scale studies and meta-analyses have demonstrated that achieving a pathological complete response (pCR) following NAC is a strong prognostic indicator for all breast cancer subtypes. In July 2020, the United States Food and Drug Administration (FDA) approved pCR as a safe endpoint for NAC^3,4^. However, NAC candidate patients should be selected carefully because there is a risk that patients who do not respond may lose their chance at surgery. Moreover, NAC consists of anti-cancer treatments that can lead to significant complications. Additionally, the residual cancer burden (RCB) index stratifies patients into distinct survival groups post-NAC, independent of pCR status^5,6^. However, in many centers, RCB is not performed as a routine test in daily practice.

Gamma-aminobutyric acid type A receptor subunit π (GABRP) is the π subunit of the GABA-A receptor and is expressed in various non-neuronal tissues^7^. GABRP, expressed via the receptor on chromosome 5q34, is thought to play a role in the development of breast, ovarian, gastric, cervical, and pancreatic cancer^8^. Studies have found that GABRP expression is significantly increased in pancreatic cancer tissues, has been associated with poor prognosis, has been found to contribute to tumor growth and metastasis, and has also been shown to have an immunomodulatory function^9^. In pancreatic cancer, GABRP causes an increase in intracellular calcium levels, and this increase activates the mitogen-activated protein kinase/extracellular signal-regulated kinase (MAPK/ERK) signaling pathway, leading to cancer progression^10^. In basal-like breast cancer cells, GABRP stimulates migration activities and expression of basal-like cytokeratins via the ERK cascade^11^. In triple-negative breast cancer (TNBC) stem cells, GABRP has been shown to interact with the epidermal growth factor receptor (EGFR) and maintain EGFR expression, resulting in maintenance of stem cell survival and chemotherapy resistance^12^.

MCM3, a subunit of MCM, is part of the multimeric Cdc45/Mcm/GINS (CMG) complex that functions as a helicase during DNA replication^13^. There are studies in the literature reporting that MCM3 is associated with treatment response in tubo-ovarian cancers^14^. Some studies investigating the role of the MCM protein family in tumor formation and progression in breast cancer have found that high expression of MCM3 is associated with treatment failure or poor prognosis, and have even identified MCM proteins as potential treatment targets^15,16^. MCM3 has been shown to be a predictive marker of response to endocrine therapy; a coordinated signaling network centered on MCM3 that limits the response to endocrine therapy in estrogen receptor (ER)-positive breast cancer has been revealed, and MCM3 has been identified as a clinically useful prognostic and predictive biomarker that allows personalized treatment of ER-positive breast cancer patients^17^.

In this study, we aimed to investigate the effects of the π subunit of the GABA-A receptor (GABRP) and MCM3 on chemotherapy responses in breast cancer and to reveal the relationship between them and the clinicopathological data of the patients. In addition, we aimed to validate the hypotheses of GABRP as a "target for drug research" and MCM-3 as a "proliferation marker".

## METHODS

### Patients

The study included patients with invasive ductal-type breast cancer who received neoadjuvant chemotherapy and then underwent surgery between March 2014 and August 2020 at Tekirdağ Namık Kemal University Hospital. Trucut biopsy samples obtained from primary breast tissue before neoadjuvant chemotherapy were used for immunohistochemical (IHC) staining of MCM3 and GABRP, Ki-67, ER, progesterone receptor (PgR), and nuclear-grade assessments. Other data were obtained from patient records.

The study included patients with human EGFR 2 (Her2)-negative tumors aged 18 years and older with sufficient tumoral tissue for trucut biopsy. All patients had invasive ductal-type histology and received standard chemotherapy. Patients who were HER2-positive, had invasive non-ductal pathology, or received chemotherapy outside of standard treatment, and patients from whom sufficient tissue samples could not be obtained for staining were not included in the study.

Clinical and pathological staging was performed according to TNM 8th Edition. Informed consent was obtained from all patients, and the study received local ethics committee approval (ethical committee protocol: 2020.230.09.17).

### Pathological assessments

Paraffin-embedded tissues and hematoxylin–eosin stained preparations previously prepared with trucut biopsy were obtained from the hospital's pathology laboratory archives, and sections were taken with a microtome by two pathology technicians. IHC staining was performed on the sections, and the sections were placed on the BenchMark XT device. Staining procedures were performed using estrogen (SP1. Ventana), progesterone (1E2. Ventana), GABRP (1:1000; ab26055; Abcam, Cambridge, UK), MCM3 (1:1000; PA1651; Boster Bio), HER-2 (anti-HER-2/neu; 4B5. Ventana), and Ki-67 (30-9. Ventana) antibodies. Normal breast tissue was used as an external control. In Her2 staining, no staining out of 3 or 1+ staining was considered negative according to the ASCO/CAP guidelines. Notably, 2+ was separated as Her2-negative and Her2-positive with fluorescence in situ hybridization (FISH)^18^.

### Statistical analysis

All statistical analyses were performed using SPSS 24 (SPSS Inc., Chicago, Ill.), and logistic regression analysis was used in multivariate and univariate model analyses to predict pathological complete response (pCR). The multivariate model was established using the "Forward: Likelihood Ratio (LR)" method. Receiver operating characteristic (ROC) analysis was used and visualized to find the ideal cut-off. The Kolmogorov-Smirnov test was used for the normality test, and Mann-Whitney U test was used for the comparison of non-normally distributed data. p-value below 0.05 was accepted for statistical significance.

## RESULTS

A total of 101 patients were included in the study, and both tumor tissues and normal breast tissues were stained using immunohistochemistry. The median age of the patients was 50 years (min: 28, max: 71). A total of 67 patients (66.3%) had luminal-type (A or B) breast cancer, while 34 patients (33.7%) had TNBC. In the pre-treatment clinical staging, 76.2% of the patients were classified as clinical T1, and 94.1% had axillary metastases. Following neoadjuvant chemotherapy, pathological complete response (pCR) was achieved in 24 patients (23.8%). The pCR rates were 39.4% in patients under 45 years of age, 38.2% in TNBC cases, and 45.2% in grade 3 tumors (Table 1).

**Table 1:** Clinical and pathological characteristics of patients and distribution of pathological complete response achieved after neoadjuvant chemotherapy.

		Total (n=101)	pCR (%) (n=24)
Age			
	<45	33	13 (39.4)
	≥45	68	11 (16.2)
Molecular subtype		0	
	Luminal A-B	67	11 (16.4)
	TNBC	34	13 (38.2)
PgR		0	
	<20	51	18 (35.3)
	≥20	50	6 (12.0)
Ki-67		0	
	<18	16	0 (0.0)
	≥18	85	24 (28.2)
Grade		0	
	Grade 1–2	59	5 (8.5)
	Grade 3	42	19 (45.2)
Clinical T stage		0	
	T1	77	21 (27.3)
	T2-T3	24	3 (12.5)
Clinical N		0	
	N0	6	2 (33.3)
	N+	95	22 (23.2)
Menopause		0	
	Pre-menopause	56	18 (32.1)
	Post-menopause	45	6 (13.3)
GABRP		0	
	<30 (median)	39	8 (20.5)
	≥30	62	16 (25.8)
MCM3		0	
	<80 (median)	47	13 (27.7)
	≥80	54	11 (20.4)

pCR: pathological complete response; TNBC: triple-negative breast cancer; PgR: progesterone receptor; GABRP: gamma-aminobutyric acid type A receptor subunit π; MCM3: minichromosome maintenance-3.

**Figure 1:**
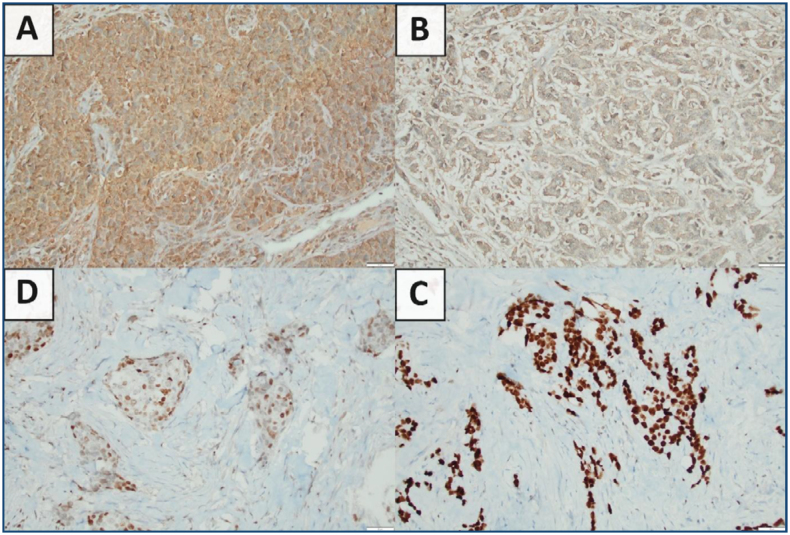
Gamma-aminobutyric acid type A receptor subunit π and minichromosome maintenance-3 staining examples [(A) minichromosome maintenance-3 Strong nuclear staining (B) minichromosome maintenance-3 Weak nuclear staining (C) gamma-aminobutyric acid type A receptor subunit π Strong cytoplasmic staining (D) gamma-aminobutyric acid type A receptor subunit π Weak cytoplasmic staining].

GABRP and MCM3 staining were performed with IHC (Figure 1A–1D). When normal breast tissue and tumor tissue of all patients were evaluated, there was no GABRP staining in normal breast tissue. In total, 97 patients (96.0%) with breast cancer expressed GABRP, and tissues of 4 patients (4%) did not show staining with GABRP (2 patients HR-positive, 2 patients TNBC). All patients had MCM3 staining (min: 1–max: 100).

When separated by median value for GABRP and MCM3, the median value for GABRP was calculated as 30% (19 patients) and the median for MCM3 as 80% (13 patients). There were 39 patients (38.6%) with a GABRP value below median, 62 patients (61.4%) with the median and above. There were 47 patients (46.5%) with MCM3 below median, 54 patients (53.5%) with the median and above value.

The mean GABRP staining percentage for all patients was 35.6±26.1%, with a mean 33.4±26.1% in HR-positive population and a mean of 39.1±26.1% for TNBC patients. Statistically both groups were similar (p=0.249). There was no correlation between GABRP expression percentage and ER, PR, and Ki-67. The mean staining percentage for MCM3 was 69.1±27.8%, 73.8±25.1 for HR-positive, and 59.9±30.6 for TNBC patients, and there was a statistically significant difference (p=0.025). There was no correlation between Ki-67 and GABRP (r=0.128, p=0.202) and MCM3 (r=-0.147, p=0.141).

When evaluated with ROC analysis, the cut-off value for pCR could not be calculated for GABRP [AUC=0.504 (95%CI 0.370–0.638), p=0.949]. The cut-off value predicting complete response could not be determined for MCM3 [AUC=0.484, (95%CI 0.354–0.614), p=0.814].

When neoadjuvant chemotherapy response was evaluated, pCR was achieved in 23.8% (n=24) of the patients. PCR was achieved in 16.4% of the patients for HR-positive and in 38.2% of the patients for TNBC. When the factors predicting pCR were evaluated by univariate analysis, age (OR 0.956, 95%CI 0.914–0.999, p=0.044), molecular subtype (OR 3.152, 95%CI 1.223–8.122, p=0.017), menopausal status (OR 0.325, 95%CI 0.116–0.906, p=0.032), Ki-67 increase (OR 1.040, 95%CI 1.019–1.063, p<0.001), ER decrease (OR 0.986, 95%CI 0.975–0.996, p<0.006), PgR decrease (OR 0.972, 95%CI 0.954–0.990, p<0.002), grade (OR 8.922, 95%CI 2.972–26.786, p<0.001) predicted treatment response. No significant association was found between treatment response and GABRP.

In multivariate analysis, nuclear grade and PgR formed a model for predicting treatment response (OR 6.309, 95%CI 2.014–19.765, p=0.002; OR 0.977, 95%CI 0.959–0.996, p=0.018, respectively) (Table 2).

**Table 2:** Univariate and multivariate logistic regression analysis of gamma-aminobutyric acid type A receptor subunit π, minichromosome maintenance-3, and other clinical and pathological parameters for complete response after neoadjuvant chemotherapy in breast cancer patients (n=101).

Variable	Category	Univariate analysis		Multivariate analysis	
		OR (95%CI)	p	OR (95%CI)	p*
Age	Continuous	0.956 (0.914–0.999)	**0.044**		
Molecular subtype	Luminal/TNBC	3.152 (1.223–8.122)	**0.017**		
ER	Continuous	0.986 (0.975–0.996)	**0.006**		
PgR	Continuous	0.972 (0.954–0.990)	**0.002**	0.977 (0.959–0.996)	**0.018**
Ki-67	<18/≥18	1.040 (1.019–1.063)	**<0.001**		
Nuclear grade	1–2/3	8.922 (2.972–26.786)	**<0.001**	6.309 (2.014–19.765)	**0.002**
Clinical T stage	T1/T2-T3	0.381 (0.103–1.411)	0.149		
Clinical N	N0/N+	0.603 (0.103–3.514)	0.574		
Menopause	Pre/Post	0.325 (0.116–0.906)	**0.032**		
GABRP	Continuous	1.001 (0.983–1.018)	0.945		
MCM3	Continuous	1.000 (0.984–1.017)	0.985		

ER: estrogen receptor; TNBC: triple-negative breast cancer; PgR: progesterone receptor; GABRP: gamma-aminobutyric acid type A receptor subunit pi; MCM3: minichromosome maintenance complex component 3; OR: odds ratio; CI: confidence interval. p*: forward-LR.

* Statistically significant p-values are marked in bold.

## DISCUSSION AND CONCLUSION

GABRP is naturally expressed in breast tissue. In breast tissue, GABRP is primarily expressed in myoepithelial/basal cells and is hypothesized to be associated with tissue contraction function^19^. In the study by Wali et al., 4,467 breast cancers were analyzed, and it was determined that GABRP was expressed at low levels in normal tissues and overexpressed in TNBC^20^. In their study, the GABRP protein was expressed in approximately half of the breast cancer tissues^20^. However, in our study, almost all (96%) of the tissues of 101 true breast cancer patients had GABRP-positive staining. This unexpectedly high positivity strengthens the potential of GABRP as a therapeutic target for both ER-positive and TNBC cases. Consistently, Wang et al. also proposed GABRP as a novel therapeutic target for TNBC^21^. The detection of GABRP expression in 32 patients with TNBC included in our study supports the hypothesis that this receptor is required for both a possible treatment target and carcinogenesis, in line with the literature.

Azuma et al.'s study on human prostate cancer cell lines demonstrated that GABA stimulation increased the production of matrix metalloproteinase (MMP) in vitro and the invasive ability of cancer cells^22^. Meng et al.'s study revealed that GABRP activates the MEK/ERK pathway, facilitates cell metastasis and tumor growth, and promotes the progression of pancreatic cancer^23^. Sizemore et al.'s study, following the seed–soil hypothesis, suggested that there may be a relationship between high GABA levels in the CNS and more frequent brain metastasis in TNBC-expressing GABRP^11^. Packard's study observed a correlation between high GABRP mRNA and preferential metastatic progression to the brain^24^. Hong et al.'s study found that upregulation of GABRP was associated with tumor cell proliferation and metastasis in the BLBC subtype^25^.

The study by Løkkegaard et al. found that upregulation of MCMs, including MCM3, was associated with resistance to endocrine therapy in patients with ER-positive breast cancer^17^. In their study, reducing MCM3 expression in breast cancer cells resistant to endocrine therapy restored drug sensitivity^17^. Zhao et al. showed that MCM3 is a proliferation marker and that there is a positive correlation with Ki-67, and that high MCM3 is a poor prognostic indicator^15^. Zou et al. concluded that low MCM3 may be a poor prognostic indicator^26^. Liu et al. showed that some MCMs may be prognostic markers, but MCM3 is not a prognostic indicator but is overexpressed in breast cancer^16^. In our study, a negative correlation was found between MCM3 and Ki-67, which was not statistically significant, and it was concluded that MCM3 expression has no effect on chemotherapy response. Indirectly, it was concluded that it is not a strong proliferation marker. However, the conclusion that low expression of MCM3 in TNBC may be a poor prognostic indicator is consistent with the studies by Zou and Liu. Many clinical factors such as heterogeneity of the cohorts and BMI may have had an impact on these results^5^.

There are some limitations in our study. The main limitation is that GABRP and MCM3 gene expressions could not be examined due to the high study cost. Another limitation is that the prognostic data of our study were immature due to insufficient follow-up, and this data was not included in the study. Another limitation was that the data obtained were retrospective and the sample group was heterogeneous. However, what makes the study strong is that we showed high GABRP in breast tumors in a real patient population, not in the estimated results from databases.

In conclusion, in our study, we showed that GABRP is expressed at a very high rate in breast cancer cells due to its molecular structure and is not expressed in normal breast tissue, and therefore, consistent with the literature, it may be a "potential target" for therapies to be developed. We also concluded that MCM3 is not a proliferation index and cannot predict treatment response, inconsistent with some previous studies. Future studies should evaluate whether GABRP expression correlates with RCB and long-term survival, as RCB has been validated as a prognostic tool in locally advanced breast cancer^6^.

## ETHICS STATEMENT

Approval no: 2020.230.09.17 (Non-Interventional Ethics Committee of Tekirdağ Namık Kemal University).

## Data Availability

The datasets generated and/or analyzed during the current study are available from the corresponding author upon reasonable request.
